# Human induced pluripotent stem cell/embryonic stem cell-derived pyramidal neuronal precursors show safety and efficacy in a rat spinal cord injury model

**DOI:** 10.1007/s00018-024-05350-9

**Published:** 2024-07-29

**Authors:** Mo Li, Boling Qi, Qian Li, Tianqi Zheng, Ying Wang, Bochao Liu, Yunqian Guan, Yunfei Bai, Fengzeng Jian, Zhi-qing David Xu, Qunyuan Xu, Zhiguo Chen

**Affiliations:** 1 Cell Therapy Center, Beijing Institute of Geriatrics, Xuanwu Hospital Capital Medical University, National Clinical Research Center for Geriatric Diseases, and Key Laboratory of Neurodegenerative Diseases, Ministry of Education, Beijing, 100053 China; 2 Center of Neural Injury and Repair, Beijing Institute for Brain Disorders, Beijing, China; 3 Center of Parkinson’s Disease, Beijing Institute for Brain Disorders, Beijing, China; 4 Department of Neurosurgery, Xuanwu Hospital Capital Medical University, Beijing, China; 5 Department of Neurobiology, Capital Medical University, Beijing, China

**Keywords:** Pyramidal neurons, Corticospinal, Induced pluripotent stem cell, Spinal cord injury, Relay circuitry, Inflammation

## Abstract

Nerve regeneration and circuit reconstruction remain a challenge following spinal cord injury (SCI). Corticospinal pyramidal neurons possess strong axon projection ability. In this study, human induced pluripotent stem cells (iPSCs) were differentiated into pyramidal neuronal precursors (PNPs) by addition of small molecule dorsomorphin into the culture. iPSC-derived PNPs were transplanted acutely into a rat contusion SCI model on the same day of injury. Following engraftment, the SCI rats showed significantly improved motor functions compared with vehicle control group as revealed by behavioral tests. Eight weeks following engraftment, the PNPs matured into corticospinal pyramidal neurons and extended axons into distant host spinal cord tissues, mostly in a caudal direction. Host neurons rostral to the lesion site also grew axons into the graft. Possible synaptic connections as a bridging relay may have been formed between host and graft-derived neurons, as indicated by pre- and post-synaptic marker staining and the regulation of chemogenetic regulatory systems. PNP graft showed an anti-inflammatory effect at the injury site and could bias microglia/macrophages towards a M2 phenotype. In addition, PNP graft was safe and no tumor formation was detected after transplantation into immunodeficient mice and SCI rats. The potential to reconstruct a neuronal relay circuitry across the lesion site and to modulate the microenvironment in SCI makes PNPs a promising cellular candidate for treatment of SCI.

**Supplementary Information:**

The online version contains supplementary material available at 10.1007/s00018-024-05350-9.

## Introduction

Spinal cord injury (SCI) is usually associated with the rupture of ascending and descending nerve tracts. Spontaneous regeneration of the injured neurons in SCI patients is very limited due to the lack of intrinsic regenerative capacity of mature neurons and the inhibitory niche at the injury site, such as glial scars and chondroitin sulfate proteoglycans (CSPGs) etc., which suppress axon growth [1].

Cell transplantation is a promising strategy to treat SCI and has a potential to regenerate damaged neurons and modulate the unfavorable microenvironment. Various types of cells have been tested that include neural stem and progenitor cells (NSPCs), mesenchymal stem cells (MSCs), Schwann cells, olfactory ensheathing cells (OECs) and oligodendrocyte precursor cells (OPCs) [2–10]. Despite decades’ research, robust long-distance regeneration of injured axons across the lesion site, and subsequent clinical improvement still remain a big challenge. During development vs. the adult stage, neurons possess a better intrinsic axon growth potential and the fetal microenvironment also provides certain neurotrophic factors that are conducive to axon growth. Induced pluripotent stem cells (iPSCs)-derived neural cells can recapitulate various stages of neural system development, and thus offer an ideal candidate graft source that could address the intrinsic and extrinsic failure of adult neuron regeneration [11, 12].

Pyramidal neurons are glutamatergic projection neurons which are characterized by a typical pyramidal morphology and a strong projection capacity. Pyramidal neurons are in charge of transmission of information between different regions of the neocortex and to other regions, such as spinal cord [13]. During development, pyramidal neurons naturally synapse onto motor neurons and interneurons; whereas motor neurons normally form synaptic connection with muscles, a non-neuronal cell type. These characteristics of pyramidal neurons led us to speculate that pyramidal neural cells may be a good cellular candidate to bridge the spinal cord lesion, possibly resulting in re-establishment of a neuronal relay circuit. In the current study, we differentiated iPSCs into pyramidal precursors and employed them in the treatment of rodent spinal cord injury models.

## Materials and methods

### Cell culture and differentiation

Human iPSCs infected with enhanced green fluorescent protein (GFP)-expressing lentivirus were expanded in E8 media and differentiated into cortical neurons according to published protocols [14]. iPSCs were dissociated into single cells and plated on matrigel on day − 2 (8 000–10 000 cells/cm^2^) in E8 medium supplemented with ROCK inhibitor. During day 0–16 of differentiation, the medium was changed to DDM [14], supplemented with B27 (10 ml B27 per 500 ml DDM, to increase survival) and Dorsomorphin; and the medium was refreshed every three days. After sixteen days in vitro (DIV), Dorsomorphin was removed from the culture medium. At twenty-four DIV, the progenitors were manually dissociated and resuspended in DDM supplemented with B27 and ROCK inhibitor (10 mM) and plated onto Poly-D-lysine (PDL)/Laminin-coated plates. Five to seven days after dissociation and replating, half of the medium was replaced with Neurobasal medium supplemented with B27 and 2 mM glutamine and changed again every five to seven days.

ESC- hM4Di-mCherry was a generous gift from Yuejun Chen’s lab at Institute of Neuroscience, Chinese Academy of Sciences. The cells were differentiated into PNPs by using the same method.

### Spinal cord injury model and cell transplantation

Thirty-six female Sprague Dawley adult rats (220–250 g; eight-ten weeks of age) were anesthetized by intraperitoneal injection of ketamine (100 mg/kg) and xylazine (10 mg/kg). A midline incision was cut to expose the spinal column at the level of T9-T12, and the paravertebral muscles were dissected bilaterally to visualize the transverse apophyses. Laminectomy was performed at T10-T11. Contusion injury was induced by using the Impact One™ Stereotaxic Impactor (Leica, Wetzlar, Germany). The parameters used for striking were as followed: 1.5 mm to the impactor tip at a speed of 1.5 m/s with a depth of 1.7 mm and a dwell of 0.1 Sect. [15]. Then the animals were randomly divided into two groups: cell transplantation group and control vehicle group. Cell suspensions or vehicle were injected at three sites, one at the middle of epicenter and the other two sites 1 mm rostral or caudal to the lesion epicenter, at a 60 degree angle, 0.5 mm lateral to the spinal midline and 1.5 mm in depth, in a total volume of either 2 µl (200 000 cells) at a rate of 1 µl/min, followed by a 3 min pause before withdrawal of the pipette. Following surgery, the rats received intraperitoneal injection of penicillin (50 000 U/kg/day; Northeastern Pharmaceutical, Shenyang first pharmaceutical Co., Ltd, CN) once a day for five days to prevent urinary tract infection. Cyclosporine A (10 mg/kg/day; Sandimmune; Novartis, East Hanover, NJ) was administered starting three days before transplantation and continued until the end of the experiments. Bladder expression was performed manually until micturition returned. The study had been reviewed and approved by the ethics committee of Xuanwu Hospital Capital Medical University. The study had been reviewed and approved by the ethics committee of Xuanwu Hospital Capital Medical University (committee’s reference number: AEEI-2015-188 and XW-20210423-2).

The motor function of rats is mainly recovered in the first two weeks after spinal cord injury, and the immune response is mainly changed greatly in the first two weeks, and scar formation occurs after two-three weeks. Therefore, the study design was divided into cell transplantation and control group, and three time points (two weeks, four weeks and eight weeks) were observed in each group, and the planned number of animals in each group was six. Thirty-six successfully modeled rats were included in the experiment, and thirty-two were reached the end of the experiment.

**Fig. 1:**
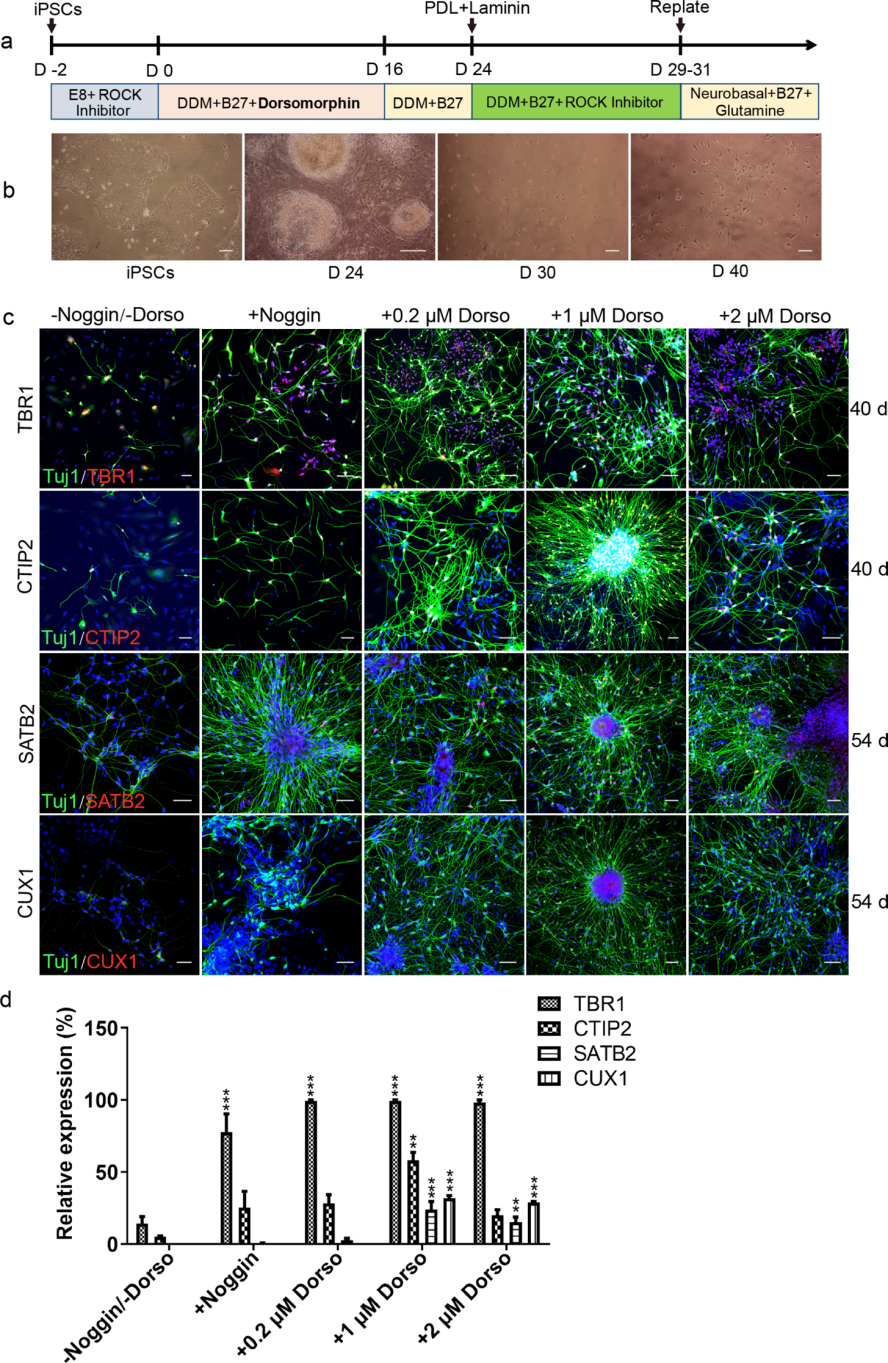
PNPs are induced from iPSCs by adding dorsomorphin. (**a**). The timeline of pyramidal cell differentiation procedure. Dorsomorphin or Noggin was added from day 0 to day 16 during differentiation. (**b**). The morphology of cells changed as iPSCs were differentiated to pyramidal neurons at different time points (scale bar, 100 μm). (**c**). Expression of markers of PNPs on differentiation day 40 and 54 with addition of different concentrations of Dorsomorphin or Noggin (Tuj1, green; TBR1/CTIP2/CUX1/SATB2, red; bar, 50 μm). (**d**). The relative expression of four transcription factors in PNPs on day 54 under different induction conditions. Data were expressed as mean ± SD (*n* = 4 for each group) (*P < 0.05; **P < 0.01; ***P < 0.001; Dorso, Dorsomorphin)

Twenty nude rats were prepared and eleven survived at the end of experiment. Five were transplanted with ESC- hM4Di-mCherry-PNPs and six with vehicle. Cell suspensions or vehicle were injected at five sites, one at the middle of epicenter and four sites 2 mm rostral and caudal to the lesion epicenter, 0.5 mm bilateral to the midline with a depth of 1 mm (Fig. 1a). Behavioral tests based on blindness.

### Open-field test

The motor function of each hind limb was assessed by Basso, Beattie, and Bresnahan (BBB) open-field locomotion test. Animals were assessed at days one, three, and seven after spinal cord injury and weekly after one week. During the three minutes of free activity in an open field, a twenty-one point scale system was used to evaluate the joint movements, stepping ability, forelimb-to–hindlimb coordination, and trunk stability. In addition, animals were recorded thirty minutes after intraperitoneal injection of CNO (Clozapine-N-Oxide, Biomol International, 4 mg/kg for inhibitory hM4Di group and control group). Each rat was recorded for three minutes within ninety minutes after CNO injection. The test was repeated twenty-four hours later and this process repeated three times.

### Ladder climbing test

A ladder with a spacing of 2 cm between steps was prepared. The ladder was tilted at an angle of 30–45 degrees, and rats were subjected to climbing from the lower side. The rats were trained to climb ladders three days prior to surgery. The total number of climbing steps and successful climbing steps were recorded each week after SCI, and three times for each time. The data were collected using successful climbing steps/total steps. The ladder climbing test with CNO administration was the same. thirty-ninety minutes after CNO injection, three repetitive climbing tests were performed on each animal.

### Immunocytochemistry

Pyramidal neurons were dissociated into single cells and plated onto coverslips coated with Poly-D-lysine and Laminin. The cells were cultured in differentiation medium containing Neural basal medium and B27. The cells were identified at different time points after thirty days. At each time point, the cells were fixed with 4% paraformaldehyde (PFA) and washed three times with PBST (0.3% Triton-X100 in PBS). The cultures were blocked with 3% donkey serum in PBST for ninety minutes at room temperature and exposed to the following primary antibodies at 4 °C overnight: rabbit anti-TBR1 (1:400, Abcam, England), rat anti-CTIP2 (1:500, Abcam), rabbit anti-CUX1/CDP (1:200, Santa Cruz, Texas, America), rabbit anti- SATB2 (1:800, Abcam), mouse anti-βIII-tubulin (1:500, Millipore, Massachusetts, America). Immunofluorescence was visualized under laser scanning confocal microscope (TCS SP5 Leica).

### Immunohistochemistry

Rats were transcardially perfused with 0.9% saline followed by 4% paraformaldehyde. Segments of spinal cord encompassing the injury site were removed and post-fixed in 4% PFA overnight at 4 °C, and then cryoprotected in 30% sucrose overnight at 4 °C. The spinal cord tissues including the injury site were embedded in OCT (802,020,001, Cellpath, England) and frozen on dry ice. Cryostat Sect. (20 μm) were cut and mounted onto gelatin-coated slides and stored at -20 °C. For immunostaining, the frozen slides were air dried at room temperature for two minutes and fixed in 4% PFA for twenty minutes. Then the sections were blocked with 3% donkey serum in PBST for two hours at room temperature, and incubated with primary antibodies overnight at 4 °C.

The following primary antibodies were used: rabbit anti-green fluorescent protein (1:1000, Invitrogen, California, American) to amplify GFP signals, goat anti-NF-L (1:100, Santa Cruz) to identify axons, rabbit-anti GFAP (1:200, Santa Cruz & 1:800, Abcam) to identify astrocytes, rabbit-anti-Homer1 (1:200, Proteintech, Illinois, American) and rabbit-anti-VGlut1 (1:200, proteintech) to identify synapse formation, goat anti-Iba1 (1:300, Abcam), biotin-anti TNFα (1:800, Xinbosheng, China), biotin-anti TGFβ1 (1:400, Xinbosheng), biotin-anti IL-1β (1:400, Xinbosheng), biotin-anti IGF-1 (1:800, R&D, New Jersey, American), rabbit-anti BDNF (1:800, Boster), mCherry (1:500, Invitrogen), Hu-Nu (1:500, Millipore). After three washes, the sections were incubated with secondary antibodies (1:200, ImmunoJackson, Pennsylvania, American) for two hours. After three washes, the sections were counterstained with DAPI and mounted. Immunofluorescence was visualized under laser scanning confocal microscope.

### Electrophysiology

#### Cell electrophysiology

Whole cell patch clamp recording was performed on iPSC-derived PNPs at day sixty of differentiation. Cells were bathed in artificial cerebrospinal fluid (126 mM NaCl, 2.5 mM KCl, 2 mM CaCl2, 1.25 mM NaH2PO4, 2 mM MgSO4, 25 mM glucose and 26 mM NaHCO3). Glass microelectrodes with intracellular liquid were used, and the electrode resistance was 3–5 MΩ. Cells were visualized by using an Olympus BX51WI system. Spontaneous action potentials and Na + K + currents were induced in current clamp mode by using a HEKA EPC-10 patch-clamp amplifier (HEKA Electronic Inc., Germany). Data acquisition and analysis were performed by using PatchMaster and Igor software (HEKA).

### MEP examination in vivo

MEP carried out for ES-DI-PNP transplantation group and control group (*n* = 5 nude rats) by using an electromyographing device (Alpine BioMed ApS, Keypoint Portable). To measure the transcranial magnetic stimulation-motor evoked potentials (TMS-MEP). After anesthesia, the stimulation electrode was placed subcutaneously 2 mm before the coronal suture and 2 mm beside the sagittal suture of animals under anesthesia. The reference electrode was placed 1 mm away from the stimulation electrode under the skin, and the ground electrode was placed under the skin at the back. The recording electrode was placed in the gastrocnemius muscle of the hindlimb. The rat cerebral cortex was stimulated with a manual monopulse square wave with intensity of 1 Hz and width of 0.1 ms, the experiment was carried out repeatedly at room temperature.

### Statistical analysis

The values of each variable (proportion of PNPs, relative abundance of individual cytokines and glia, and BBB scores) were calculated by using GraphPad Prism 5 (Graphpad software, La Jolla, CA). The data were expressed as Mean ± SD. Statistical analysis was carried out by using SPSS16.0 (IBM, Armonk, NY, USA). Different groups were compared by using ANOVA. A value of *p* < 0.05 was considered statistically significant.

## Results

### Differentiation of iPSCs into PNPs induced by noggin and dorsomorphin

It has been reported that iPSCs can be differentiated to PNPs through employment of a small molecule ‘Noggin’ [14]. In the present study, we used another small molecule ‘Dorsomorphin’ that has similar functions to noggin for PNP differentiation. ‘Dorsomorphin’ can mediate BMP inactivation by selectively inhibiting BMP type I receptors ALK2 ALK3 and ALK6 [16]. The process of differentiation was shown in Fig. 1a. We added different concentrations of dorsomorphin (0.2 µM, 1 µM and 2 µM) or 1 µM noggin (as control) from day one to day sixteen during the differentiation process to compare the effects of dorsomorphin vs. noggin. Changes in cell morphology could be seen at different time points of differentiation. The PNPs started to appear from differentiation day thirty, and neurons with obvious processes were observed from day forty (Fig. 1b and c). The results showed that iPSCs could be differentiated into pyramidal neurons by adding dorsomorphin. The resultant cells were positive for pyramidal neuron markers TBR1, CTIP2, CUX1 (positive in 1 µM and 2 µM dorsomorphin groups), SATB2, and pan neuron marker β-tubulin III (Tuj1) (Fig. 1c). The relative expression levels of the four transcription factors were higher in 1 µM dorsomorphin vs. the other concentrations (Fig. 1d). Therefore, 1 µM dorsomorphin was chosen for the rest of the differentiation experiments in this study.

### Differentiation of iPSCs into functional PNPs with 1 µM dorsomorphin

**Fig. 2:**
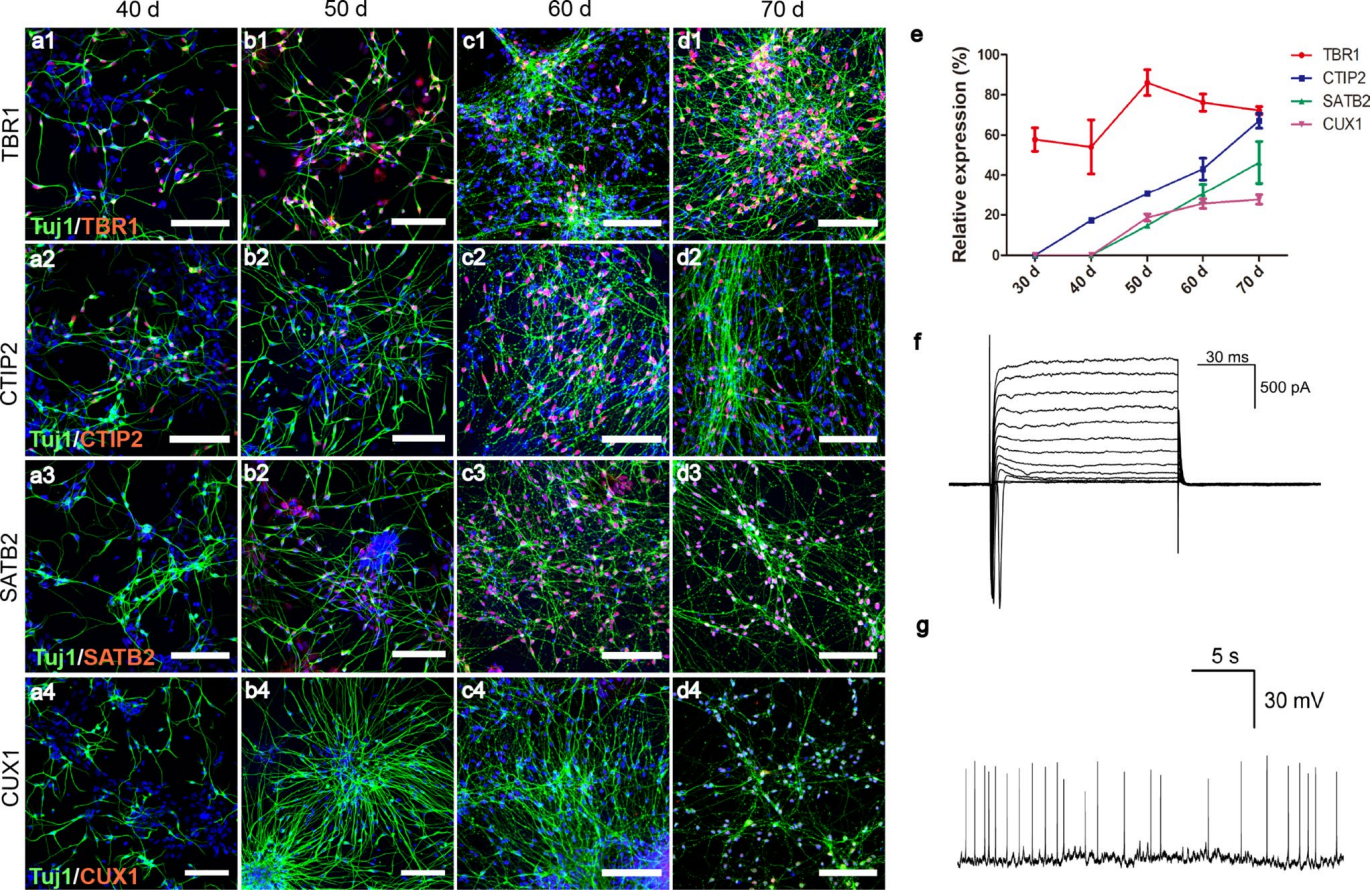
Identification of PNPs induced by Dorsomorphin. (**a**-**d**) Expression of PNP markers at different time points. (Tuj1, green; TBR1/CTIP2/CUX1/SATB2, red; bar, 50 μm); (**e**). The proportion of cells that expressed different pyramidal cell markers at different time points. Data were expressed as mean ± SD (*n* = 4 for each group). (**f**). Detection of sodium and potassium channels with voltage holding from − 60 to + 60 mV. (**g**). Spontaneous action potentials were detected under the holding at resting current

The expression of four transcription factors related to development of pyramidal neurons were characterized on differentiation days forty, fifty, sixty and seventy (Fig. 2a-d). TBR1-positive cells were detected by day thirty, CTIP2 expression started to take place at around day thirty-five to forty, when only a few dim SATB2 and CUX1 signals could be detected. With time, the number of cells positive for each marker, TBR1, CTIP2, SATB2, or CUX1, increased gradually, and the intensities of SATB2 and CUX1 signals were also enhanced. Quantitative analysis of immunofluorescent staining revealed that, the proportions of TBR1 + cells remained relative high (60–90%) from day thirty to day seventy, and the percentages of cells positive for CTIP2, SATB2, or CUX1 gradually increased over time (Fig. 2e). The temporal order of marker expression was in agreement with the development of cerebral cortex, with deeper layer cells that expressed TBR1 appearing first, followed by relatively upper layers positive for CTIP2, SATB2 and/or CUX1 (Fig. S1) [17]. CTIP2 (COUP-TF interacting protein 2) is a zinc finger protein that is associated with the extension, fasciculation and path-finding of subcortically projecting neurons. Studies showed that, in CTIP2 null mice, corticospinal axons fail to extend beyond the pons [18]. CTIP2-positive neurons mostly correspond to layer V corticofugal neurons that project to the midbrain, hindbrain, and spinal cord [14, 19]. By differentiation day fifty, around 55% of cells were positive for CTIP2, and some of the CTIP2 + cells were negative for TBR1 (Fig. S2a-d). Cells of this stage could possess long distance projection ability and meanwhile retain certain proliferative capacity, which might be an ideal candidate for transplantation studies to treat SCI. The cells differentiated for sixty days were subjected to patch clamping. Potassium and sodium currents and spontaneous action potentials were detected, indicative of a mature neuronal stage (Fig. 2f-g). Presynaptic marker of glutamatergic neurons, VGlut1, and postsynaptic marker, Homer1 were also detected by differentiation day sixty (Fig. S2e-l).

### PNPs show axon projection ability following transplantation into immunodeficient mice

**Fig. 3:**
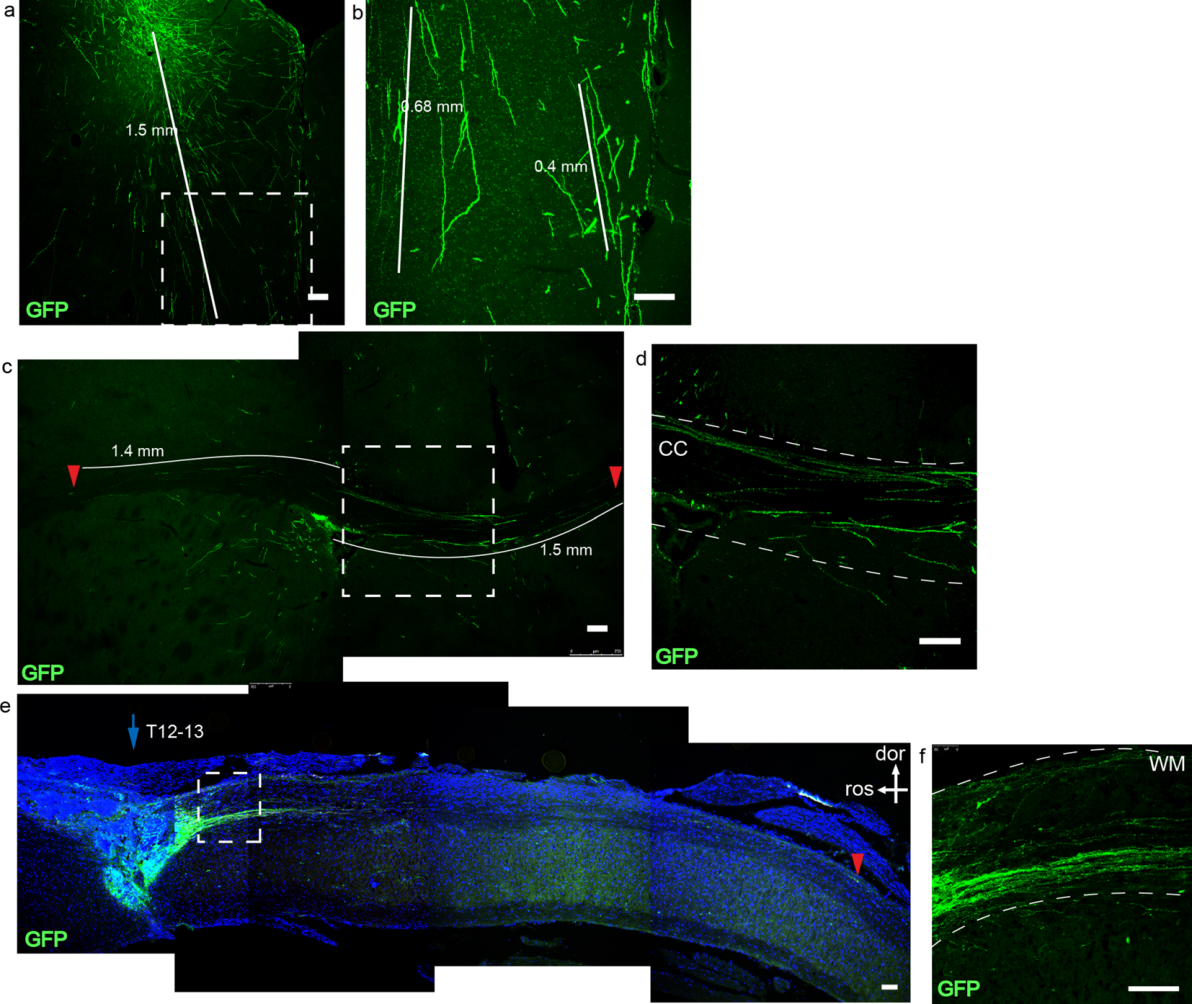
Transplantation of PNPs into immunodeficient mice. (**a**). Two months after transplantation of PNPs into the brains of immunodeficient mice. Engrafted PNPs in cerebral cortex projected axons downward to the deeper layers of the brain. (**b**). The amplified area of the white box in figure a, which highlighted the projected axons. (**c**). Axon projection two months after transplantation of PNPs into the corpus collosum. The red arrow indicated the end point of a detected axon. (**d**). The area outlined in the white box in figure c. CC, corpus callosum. (**e**). One month after transplantation of PNPs into the spinal cord. Axons projected along the white matter of the spinal cord. The red arrow head indicated the end of a long-projected axon, which was about 4.5 mm from the engraftment site. Figure f was the magnified view of the inset in e. (dor, dorsal; ros, rostral; WM, white matter). Bar, 100 μm

During development, pyramidal neurons can form corticospinal tract that covers a long distance, suggesting that pyramidal neurons possess a robust axon projection ability - a feature that could be used as a bridging tool for neuronal circuit reconstruction after SCI. Next, we investigated whether the obtained PNPs can project axons after transplantation into naïve immunodeficient mice. GFP-labeled iPSCs were differentiated for fifty days and the resultant PNPs were transplanted into the cerebral cortex, corpus callosum and spinal cord of immunodeficient mice, and were examined one month and two months later (Fig. 3). Two months after transplantation, surviving cells were detected at the injection sites. Cells engrafted at the cerebral cortex were detected at and around the bolus, which projected axons downward into the deeper part of the brain. The GFP + axons covered a distance of up to 1.5 mm (Fig. 3a and b). At the corpus callosum, GFP + axons were detected along the nerve fiber bundle, and some axons covered a distance of up to 1.5 mm (Fig. 3c and d).

PNPs could also survive and project axons after transplantation into the spinal cord (T12-13) of naïve immunodeficient mice. One month after transplantation, the axons mainly projected along the white matter of the spinal cord, spanning a distance of up to 4.5 mm (Fig. 3e and f). The results demonstrated that PNPs of differentiation day fifty could survive transplantation and displayed a strong axon projection ability.

### PNP engraftment improves the motor function of SCI rats

**Fig. 4:**
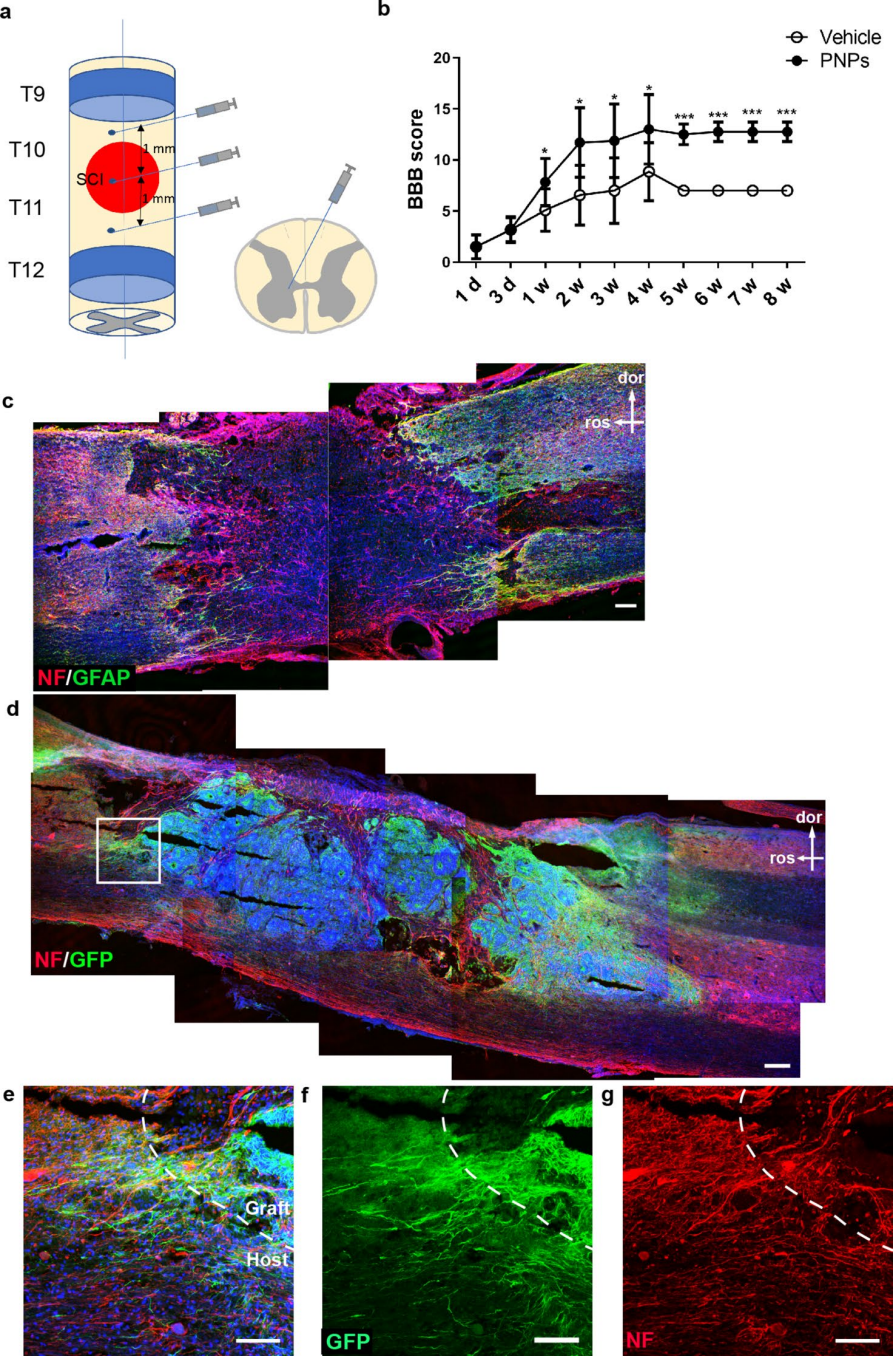
Motor function and pathological changes following transplantation of PNPs into SCI rats. (**a**) Schematic representation of epicenter (red area) and transplanted sites (three sites of injection). (**b**) BBB scores in SCI rats after transplantation with PNPs or vehicle. The values were presented as means ± SD (*n* = 5; * *P* < 0.05; ** *P* < 0.01; *** *P* < 0.001; d, day; w, week). (**a**-**d**) Staining on spinal cord sections from vehicle (**c**) and PNP (**d**) groups two weeks after SCI. In the vehicle group (**c**), neural fibers were discontinued at the lesion site and GFAP + astrogliosis was detected at both sides of the lesion. With PNP engraftment (**d**), some remaining neurofilament-positive fibers were observed close to the dorsal and ventral boundary of the spinal cord, and the GFP-positive grafts occupied the lesioned space and extended axons along the spinal cord (**e**, **f**). Meanwhile, host neurons might also extend axons across the host-graft interface into the graft (**g**). dor, dorsal; ros, rostral; bars in d and d, 200 μm; bar in e-g, 100 μm

The SCI contusion model was established by dropping an impactor onto the thoracic vertebrae ten-eleven (T10 - T11) of Sprague Dawley rats. Rats with complete paraplegia received either PNP engraftment or buffer only, in the same day immediately after SCI (Fig. 4a). The Basso, Beattie and Bresnahan (BBB) locomotor scales were evaluated at day one, day three, and every week afterwards. The SCI rats in cell transplantation group showed significantly improved motor performance from one week post-engraftment onward compared with the vehicle group (Fig. 4b).

**Fig. 5:**
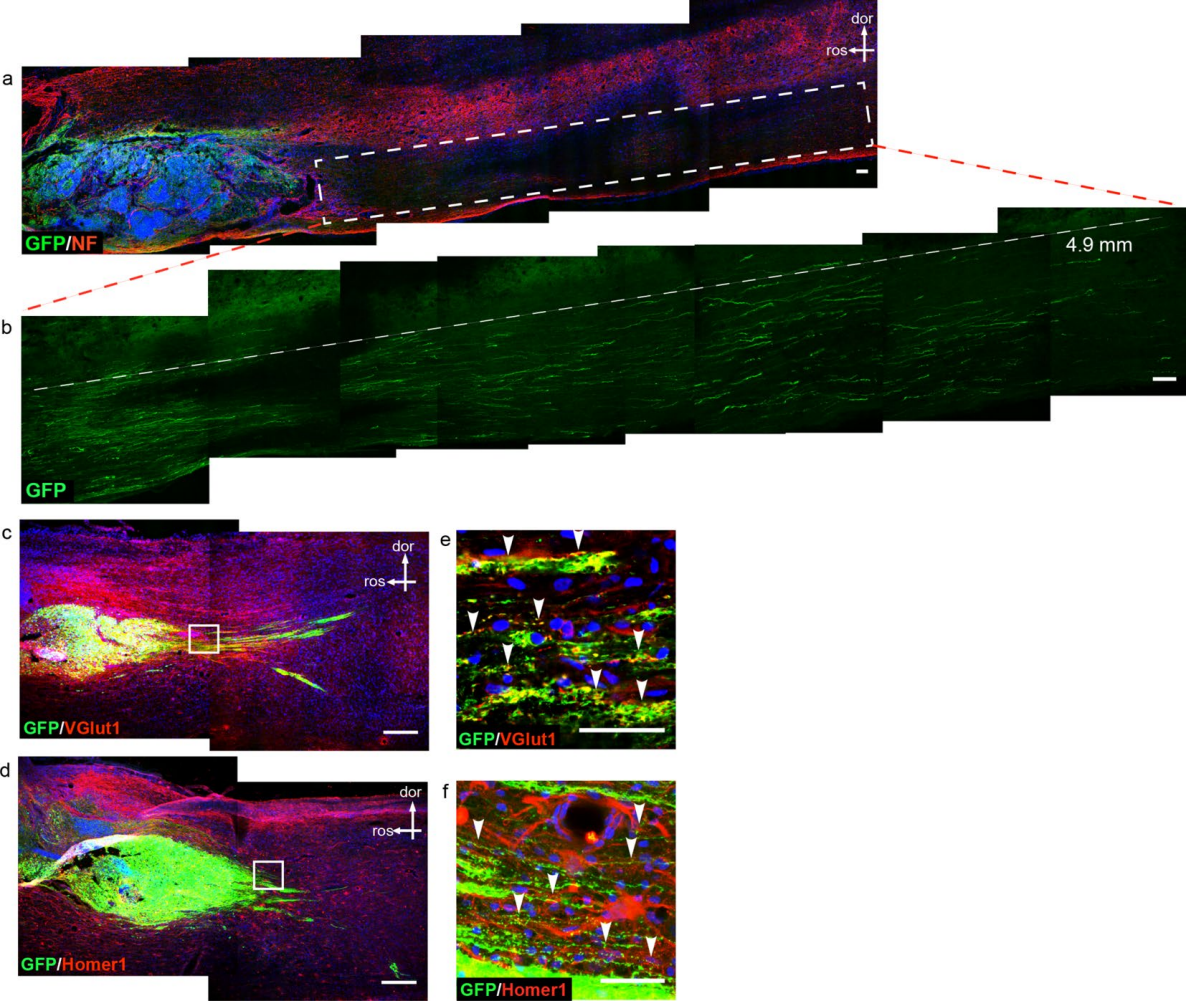
The axons of the transplanted cells extend into the host spinal cord and express synaptic markers. (**a**). GPF-labeled graft-derived axons extended into the host spinal cord and projected into distant areas. Figure (**b**). represented the magnified view of the white box in **a**. (**c**, **e**) GFP-positive axons were co-labeled with presynaptic glutamatergic marker Vglut1(red). (**d**, **f**). GFP-positive axons were juxtaposed to postsynaptic marker Homer1 (red), as indicated by the arrow heads. dor, dorsal; ros, rostral; bar, 100 μm in **a** and **b**; bar, 250 μm in **c** and **d**; 25 μm in **e** and **f**

Two weeks following contusion injury, all of the rats in the injury group receiving vehicle only showed completely discontinued cords, with the nerve tract broken from the dorsal to the ventral part around the center of impact (Fig. 4c). In contrast, SCI rats with PNP engraftment showed a certain degree of tract sparing at the dorsal and ventral edges of the spinal cord (Fig. 4d), implying that engraftment might have ameliorated the secondary damage during the acute stage following SCI. Some axons were detected that emanated from the graft and grew into the host spinal cord, and there were also GFP-/NF + host rat axons detected that grew into the graft (Fig. 4e-g). One month after transplantation of PNPs, the graft-derived axons extended into the host tissues and projected along the spinal cord fasciculi, mainly in a caudal direction, which covered a distance of up to 4.9 mm (Fig. 5a and b).

### Possible reconstruction of relay circuit between PNPs and host neurons

One possible reason accounting for the improved motor functions was the reconstruction of neuronal circuit between grafted cells and host neurons. Two months after transplantation, axon fibers were detected which emanated from the graft and grow into the host spinal cord tissue (Fig. 5c and d). In the area caudal to the transplantation site, GFP + axons were found co-labeled with presynaptic marker VGlut1 (Fig. 5e), and some GFP + axons were juxtaposed to postsynaptic marker Homer1 (Fig. 5f). At the caudal interface between host tissue and graft, GFP-/NF + host axons were detected that grew into the graft (Fig. S3). The results suggested that graft-derived pyramidal neurons might have established synapses with host neurons.

**Fig. 6:**
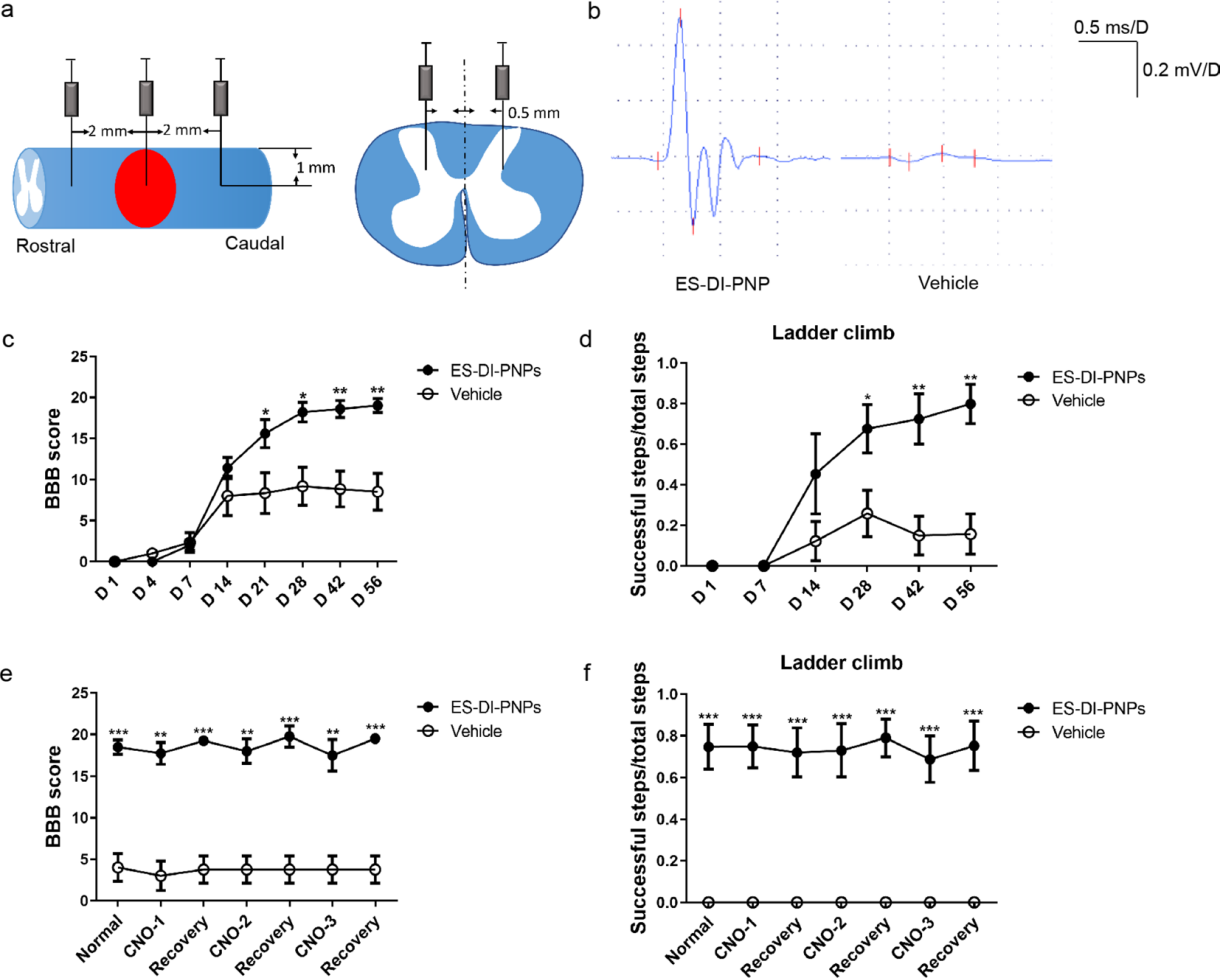
Locomotor recovery in nude rats following cell transplantation. (**a**). Transplanted sites of ESC-DI-PNPs in nude rats. (**b**). Evoked action potentials were observed following engraftment of PNPs. (**c**). BBB score of SCI nude rats with or without transplantation of ESC-DI-PNPs. (**d**). Results of ladder climbing test. (**e**, **f**). Results of BBB and ladder climbing tests following CNO administration in transplantation and vehicle groups. (*n* = 5; *P < 0.05; **P < 0.01; ***P < 0.001; compared with vehicle groups)

**Fig. 7:**
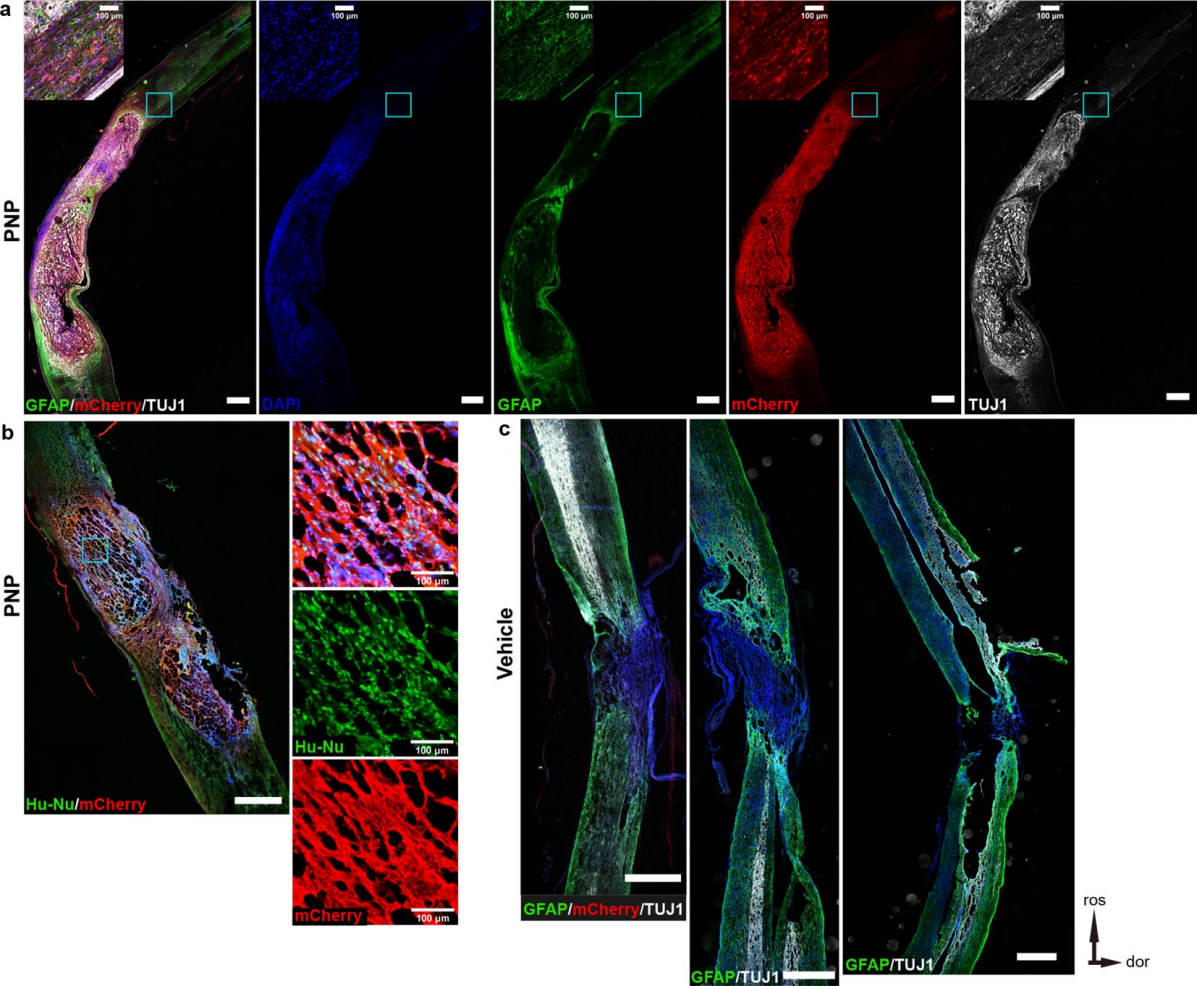
Histological analyses demonstrating transplanted cells can repair damaged spinal cord. (**a**) Immunofluorescence staining of the spinal cord tissue of nude rat following engraftment of ESC-DI-PNPs (bar, 1 mm). (**b**) PNPs survived in vivo (Hu-Nu, Human Nuclei). (**c**) The spinal cords were completely severed in vehicle control group (bar, 1 mm). dor, dorsal; ros, rostral

To further verify the relationship between transplanted PNPs and host neurons, we used a CNO-regulated ESC cell line with a chemogenetic regulatory system. Figure 6a shows the cell transplantation sites. CNO treatment abrogated spontaneous action potentials in the inhibitory hM4Di-expressing PNPs. The results showed that, after transplantation of ESC- hM4Di-mCherry-PNPs, the MEP testing evoked action potentials were detected in the transplantation group (Fig. 6b), and the locomotor function of nude rats recovered to a better degree than control group (Fig. 6c and d). BBB and ladder climbing test revealed a slightly decreased motor function 30 min following intraperitoneal injection of CNO, which recovered twenty-four later (Fig. 6e and f). Nevertheless, the scores with CNO treatment were not statistically significant compared with pre- or post-CNO treatment scores. Immunofluorescence staining showed that the spinal cord of nude rats in the cell transplantation group displayed a higher degree of integrity with a large number of PNPs surviving (Fig. 7a and b), while the spinal cord in the control group was completely severed with an obvious cavity (Fig. 7c). Following cell transplantation, some nerve fiber bundles were observed at the dorsal and ventral sides of the injured spinal cord. These results indicate that functional connection between PNPs and host neurons might have been reconstructed.

### Gliosis at the lesion site

**Fig. 8:**
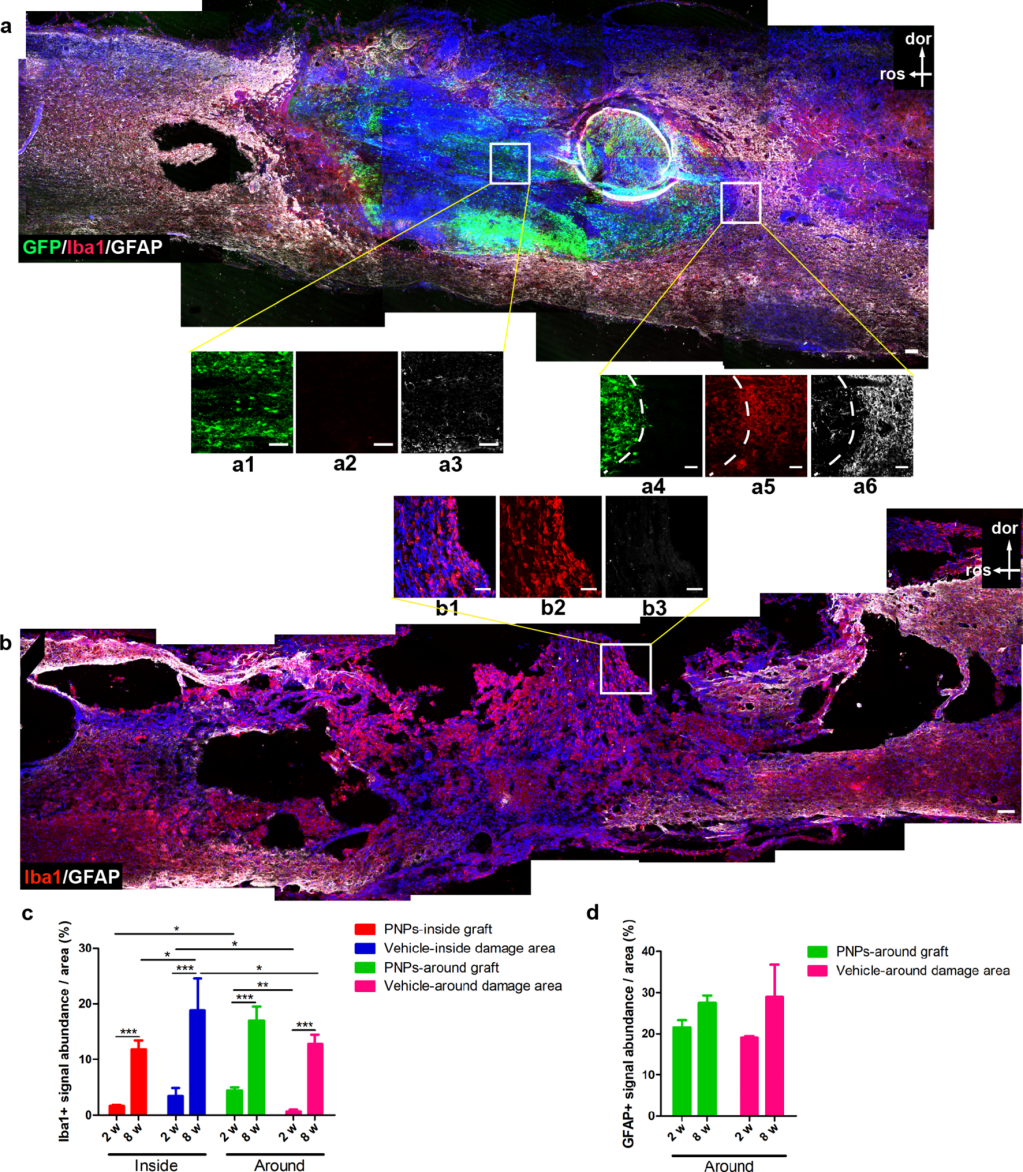
Gliosis at the lesion site two months after SCI with or without transplantation. (**a**, **b**) Staining results of microglia/macrophages (Iba1) and astrocytes (GFAP) in PNP group (**a**) and vehicle group (**b**) two months after SCI. (**c**, **d**) Quantification of Iba1 + signals (**c**) and GFAP + signals (**d**) at different areas of the lesion sites. *n* = 5; * *P* < 0.05; ** *P* < 0.01; *** *P* < 0.001. Iba1, red; GFAP, white; d, dorsal; r, rostral; bar, 100 μm in a and b; 50 μm in a1-a6 and b1-b3

Next we examined the level of gliosis inside and around the lesion at different time points (Fig. 8a-d). Although the rats had been administered cyclosporine as an immunosuppressant, the immune functions may not be completely suppressed, particularly the innate immune functions may be spared. At two months post-transplantation, a significantly lower number of Iba1 + microglia/macrophages were detected inside the graft in the transplantation group, vs. inside the lesion of the vehicle group (Fig. 8a-c). At two months, there were more Iba1 + cells observed inside the lesion site vs. around the lesion in the vehicle group (Fig. 8c). At two weeks post-transplantation, fewer Iba1 + cells were detected inside and around the lesion, compared with the two months time point, suggesting that microglia/macrophages may have accumulated over time at the lesion site. Interestingly, at two weeks post-transplantation, there were fewer microglia/macrophages inside the graft vs. at the graft/host interface (Fig. 8c). In contrast, in the vehicle group, more macroglia/macrophages were observed inside the lesion vs. around the lesion (Fig. 8c). The results indicated that the transplanted PNPs might have had an effect of suppressing Iba1 + cells. Regarding astrogliosis, few GFAP + cells were observed inside the lesion at two weeks and two months (data not shown); and GFAP + signals were detected around the lesion, but no significant difference existed between the different groups (Fig. 8d).

Microglia/macrophages can be categorized into M1 and M2 phenotypes [20]. M2 microglia/macrophages are able to express IGF1 and TGF-β1, and are generally considered to play a neurotrophic and anti-inflammatory role. We stained for IGF1 and TGF-β1 on spinal cord sections from two weeks groups, and detected a significant number of Iba1 + cells that co-expressed IGF1 or TGF-β1 (Fig. S4). But such cells were rarely observed in the vehicle group (data not shown). The results suggested that engrafted PNPs might have influenced microglia/macrophages and biased them towards a M2 phenotype.

We also investigated the expression of TNFα, IGF1 and BDNF at the lesion site at two weeks, four weeks, and eight weeks post-transplantation (Fig. S5a). We measured the cell densities positive for each of the above molecule in an area at the lesion boundary (about 1 mm rostral and 1 mm dorsal from damage edge). TNFα + cells were higher at two weeks in graft group vs. vehicle group, possibly due to the immune recognition of xenograft. But the TNFα + cells gradually decrease over time in graft group and increase in the vehicle group (Fig. S5b). More IGF1 + cells were also observed at two weeks in graft group vs. vehicle group (Fig. S5c). No BDNF + cells were detected at eight weeks post-transplantation. But from two weeks to four weeks, BDNF + cells increased in the engraftment group but decreased in the vehicle group (Fig. S5d).

## Discussion

In the current study, we differentiated iPSCs/ESCs into PNPs by addition of 1 µM dorsomorphin, and transplanted the PNPs into a rodent SCI model. Behavioral analysis revealed an improved motor function in the transplanted animals vs. vehicle group. Engraftment of PNPs in SCI animals also alleviated the pathology at the lesion site. After transplantation, the PNPs matured into pyramidal neurons and extended axons into host spinal cord tissue, mostly in a caudal direction. Receiving synaptic connection from supraspinal neurons and forming downstream synapses with the host neurons caudal to the lesion might reconstruct the neuronal relay circuit, leading to restoration of motor function. This was also demonstrated by using chemogenetically engineered PNPs. The PNP graft could also modulate the microenvironment at the lesion site by producing neurotrophic factors and/or biasing inflammatory and anti-inflammatory balance.

The key to curing SCI is to establish the neuronal relay across the lesion site, so that the voluntary signals descending from the cortex could be transmitted through the lesion to control the muscles, and the sensory information be transmitted from the periphery to the brain. One way to build the relay is to regrow the spared host corticospinal axons and/or the host intraspinal interneuron axons across the lesioned area and form new synaptic connections beyond the lesion with host neurons. However, this approach is challenging in two folds. First, the majority of clinical SCI are complete, leaving little spared tissue at the lesion site. Second, components on myelin and astrocytic scar tissues in the adult spinal cord are inhibitory to axon regrowth. Liu et al. reported that deletion of phosphatase and tensin homolog (PTEN) endows the remaining axons with a capacity to resist the inhibitory signals at the lesion site [20, 21]. However, genetic editing on the host spinal cord to treat SCI still face many challenges and has a long way to go [22]. The other way to build the relay is to transplant neural tissues to the lesion cavity. When the neural tissues mature to neurons and make synaptic connections with the upstream and downstream host neurons, a relay circuit can be established, possibly leading to functional outcome. Tuszynski group employed human fetal spinal cord tissue-derived neural stem/progenitor cells for transplantation into a nonhuman primate hemisection SCI model. The graft can differentiate to astrocytes and neurons; graft-derived neurons project axons into host tissues and host neurons also grow into the graft-occupied cavity, consistent with an improved forelimb function [23]. The study has led to a clinical trial in 2018 [24]. Fetal spinal cord-derived neural stem/progenitor cells normally give rise to pan-neurons – a mixture of neuronal subtypes, and glial cells [7]. Fine control of motor and sensory functions requires sophisticated and precise orchestration of neuronal network/circuitry. Integration of only specific neuronal subtypes into the damaged circuitry would be assumed to be a requirement for optimal clinical readout and avoiding possible side effect, although what types of cells should be added into the network remain elusive. To date, the types of nerve cells that can be used in SCI therapy are limited, which included human fetal spinal cord tissue-derived neural stem/progenitor cells (NSI-566), human CNS Stem Cells (CNS-SCs), and H1 human Embryonic Stem Cell (hESC) line-derived Oligodendrocyte Progenitors Cells (OPCs, AST-OPC1) [3, 25]. Finding a suitable alternative neuronal type for treatment of spinal cord injury remains warranted.

A substantial amount of spinal cord neuronal connections are made between corticospinal neurons and lower motor neurons. An ideal candidate would possess a good axon projection ability and could receive synaptic connection from upstream corticospinal axons and be ready to synapse onto downstream motor neurons. Lower motor neurons have good axon projection ability but naturally synapse onto muscles. A neuron-muscle synapse is structurally different from a neuron-neuron synapse. Therefore, we chose to put in the upper corticospinal neurons into the lesioned cord as a bridging relay in the current study. Pyramidal neurons located at the cortex can naturally send long-distance axonal projection to synapse onto the motor neurons in the spinal cord. The strong axonal extension and projection capacity may be conducive to reconstructing the functional connection bridging the lesion area in SCI. Furthermore, compared with motor neurons as tested in some preclinical studies, pyramidal neurons may be more predisposed to synapsing onto the downstream motor neurons in the injured spinal cord.

iPSC-derived PNPs matured and extended axons into distant areas when transplanted into rodent brain cortex, corpse collosum, and spinal cord, showing a strong intrinsic axon projection ability, even in the inhibitory niche of adult nervous system (Figs. 3, 4 and 5). After engraftment into the contusion-induced lesion area, PNPs differentiated into mature neurons and received incoming synaptic connections from host neurons rostral to the lesion (Fig. 4d-g). Graft-derived neurons also extended axons to the distant host spinal cord tissues, mostly in the caudal part to the lesion (Fig. 5). The synaptic connections with host cells were suggested by labelling of presynaptic marker VGlut1 and postsynaptic marker Homer1 (Fig. 5), and was also indicated by MEP tests and by using chemogenetically engineered PNP graft.

Safety is an important consideration related to cell therapy, particularly when pluripotent stem cells-derived cells are involved. iPSC-derived PNPs were transplanted to different parts in immunodeficient mice, and no tumor formation was detected two months later, suggesting that the cells might be safe. Nevertheless, using iPSC-derivatives as a graft may need to go through more strict safety measures to eradicate any remaining tumorigenic cells before such a strategy could meet regulatory requirements and be considered in clinical trials.

We applied a contusion SCI model in the current study due to the relevance of this model to clinical conditions. The PNPs were injected the same day of contusion-induced injury to mimic an acute therapeutic approach, and also in an attempt to reduce the suffering of animals in a second open-surgery if injected at a later time point. However, this treatment time window (same day of SCI) may not be easily realized in clinical practice. Further studies using a later time point of cell therapy are warranted. In addition, there are obvious differences in the functional neuroanatomy between rodents and primates. Many therapies that worked on rodents failed in clinical trials. Applying PNPs in a nonhuman primate SCI model may serve as a translational step towards possible future clinical application.

Not only showing a potential to construct a relay circuit, PNPs also seemed to be able to modulate the microenvironment at the lesion site. At two weeks post-engraftment, the number of Iba1 + microglia/macrophages were lower at the interior part of graft, compared with the graft/host interface area, and also lower than the interior part of lesion in the vehicle control group (Fig. 8). The results suggest the PNP graft may possess some anti-inflammatory property. Interestingly, PNP graft seemed to be able to bias microglia towards a IGF1- and TGF-β1-positive M2 phenotype, which was not seen in the vehicle group without engraftment. With PNP transplantation, the lesion areas also presented with a higher level of trophic factors, such as IGF1 and BDNF (Fig. S5). This niche-modulating effect could result from the fetal development stage-resembling feature of iPSC-derived PNPs, and/or a specific function of this particular cell type - PNPs. The anti-inflammatory and trophic effect may reduce the degree of secondary damage following SCI and/or promote graft survival and maturation, eventually leading to a functional recovery. Further studies are needed to investigate the molecular mechanisms of such an effect.

Several strategies have been investigated for treatment of spinal cord injury. For example, application of biomaterials has shown a significant effect, particularly in the presence of tissue cavities caused by lesion itself or by removal of lesion scars [26–28]. Some studies also pointed out that stem cells combined with rehabilitation or materials are more beneficial to the repair of spinal cord injury [29]. Recently, the research using spinal-cord organoids has also made some preliminary progress [30]. It is possible that a combinatory approach might offer a more efficacious strategy for treatment of spinal cord injury.

## Conclusions

In this study, we used iPSC/ESC-derived pyramidal precursor cells for treatment of SCI. PNPs had a remarkable effect on behavioral recovery following severe spinal cord injury, and the motor function of the animals were restored significantly. PNPs played a role in tissue repair and formed synaptic connections with the host neurons. PNPs also provided anti-inflammatory effects, and promoted the transformation of microglia into an M2 phenotype. The results may pave a road to future clinical translation for treatment of SCI patients.

### Electronic supplementary material

Below is the link to the electronic supplementary material.

Supplementary Material 1

## Data Availability

The datasets generated during and/or analysed during the current study are available upon request from the corresponding author.

## Abbreviations

iPSCs: Induced Pluripotent Stem Cells
PNPs: Pyramidal Neuronal Precursors
CSPGs: Chondroitin Sulfate Proteoglycans
NSPCs: Neural Stem and Progenitor Cells
MSCs: Mesenchymal Stem Cells
OECs: Olfactory Ensheathing Cells
OPCs: Oligodendrocyte Precursor Cells
GFP: Green Fluorescent Protein
BBB: Basso, Beattie, and Bresnahan
CNO: Clozapine-N-Oxide
PTEN: Phosphatase and Tensin homolog
