# Supplementary material for: Human induced pluripotent stem cell/embryonic stem cell-derived pyramidal neuronal precursors show safety and efficacy in a rat spinal cord injury model
Source: Cell Mol Life Sci. 2024 Jul 29;81(1):318. doi: 10.1007/s00018-024-05350-9 (PMC11335242; doi:10.1007/s00018-024-05350-9)
Supplement: Supplementary file 1 — Supplementary Material 1 [file 18_2024_5350_MOESM1_ESM.docx]

Human induced pluripotent stem cell/Embryonic stem cell-derived pyramidal neuronal precursors show safety and efficacy in a rat spinal cord injury model

**Running title: PNPs to treat spinal cord injury**

Mo Li^1,2,3,4^, Boling Qi^1,2,3^, Qian Li^1,2,3,4^, Tianqi Zheng^1,2,3^, Ying Wang^2,5^, Bochao Liu^1,2,3^, Yunqian Guan^1,2,3^, Yunfei Bai^2,5^, Fengzeng Jian^4^, Zhi-qing David Xu^2,5^, Qunyuan Xu^2,5^, Zhiguo Chen^1,2,3*^

^1^Cell Therapy Center, Beijing Institute of Geriatrics, Xuanwu Hospital Capital Medical University, National Clinical Research Center for Geriatric Diseases, and Key Laboratory of Neurodegenerative Diseases, Ministry of Education, Beijing 100053, China

^2^Center of Neural Injury and Repair, Beijing Institute for Brain Disorders, Beijing, China

^3^Center of Parkinson's Disease, Beijing Institute for Brain Disorders, Beijing, China

^4^Department of neurosurgery, Xuanwu Hospital Capital Medical University, Beijing, China

^5^Department of Neurobiology, Capital Medical University, Beijing, China

*** Correspondence:**Zhiguo Chen
[chenzhiguo@gmail.com](mailto:chenzhiguo@gmail.com)

**Acknowledgements**

Yuejun Chen’s lab was thanked for the generous gift of chemogenetic ESC lines.

Supplementary Material

Figure S1………………………………………………………………………………………………2

Figure S2………………………………………………………………………………………………3

Figure S3………………………………………………………………………………………………4

Figure S4………………………………………………………………………………………………5

Figure S5………………………………………………………………………………………………6


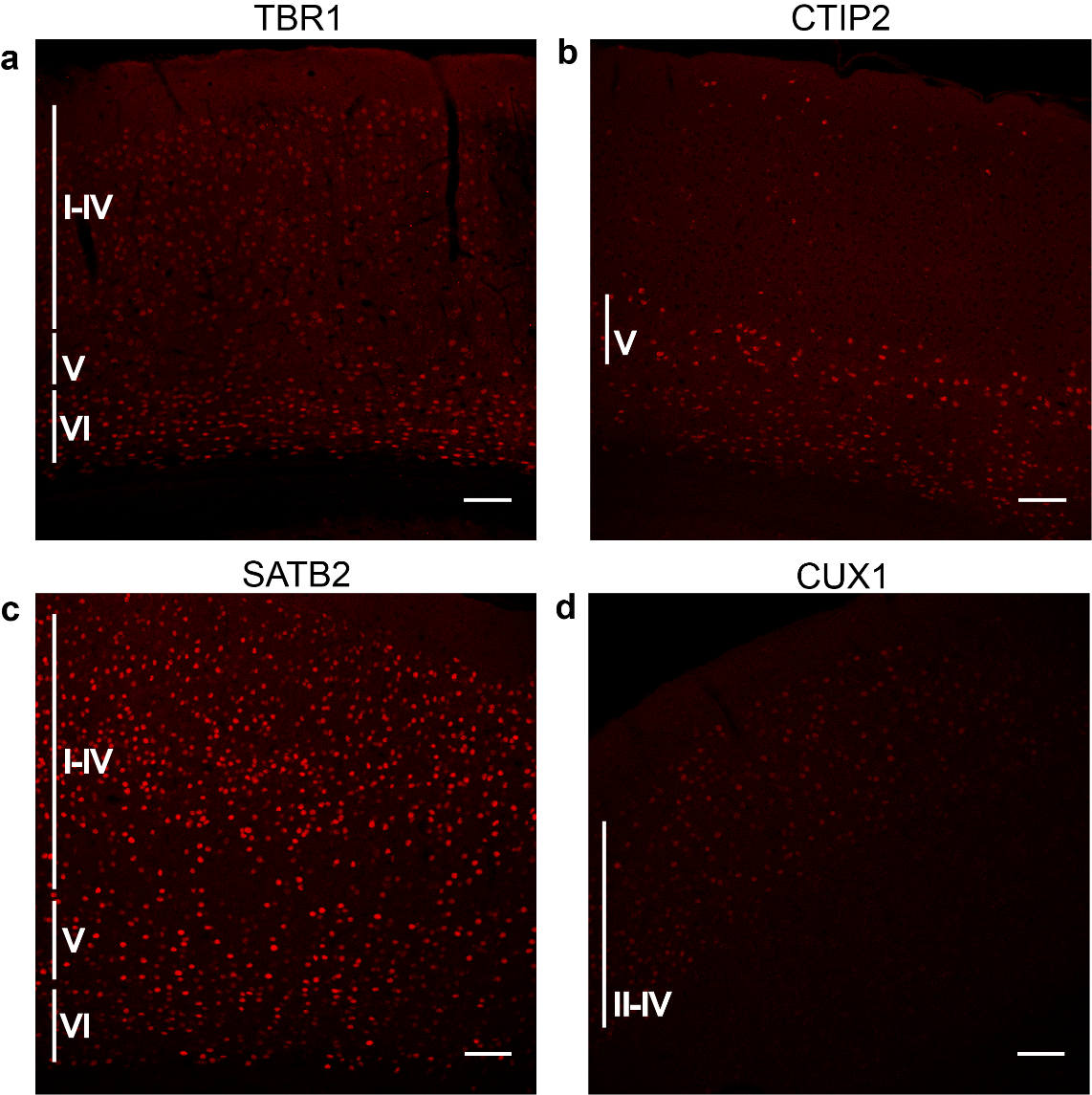


**Figure S1 The distribution of pyramidal neurons in the mouse cerebral cortex.** (a-d) The expression of TBR1, CTIP2, SATB2 and CUX1 in pyramidal neurons at different cortical layers. Bar, 75 μm.


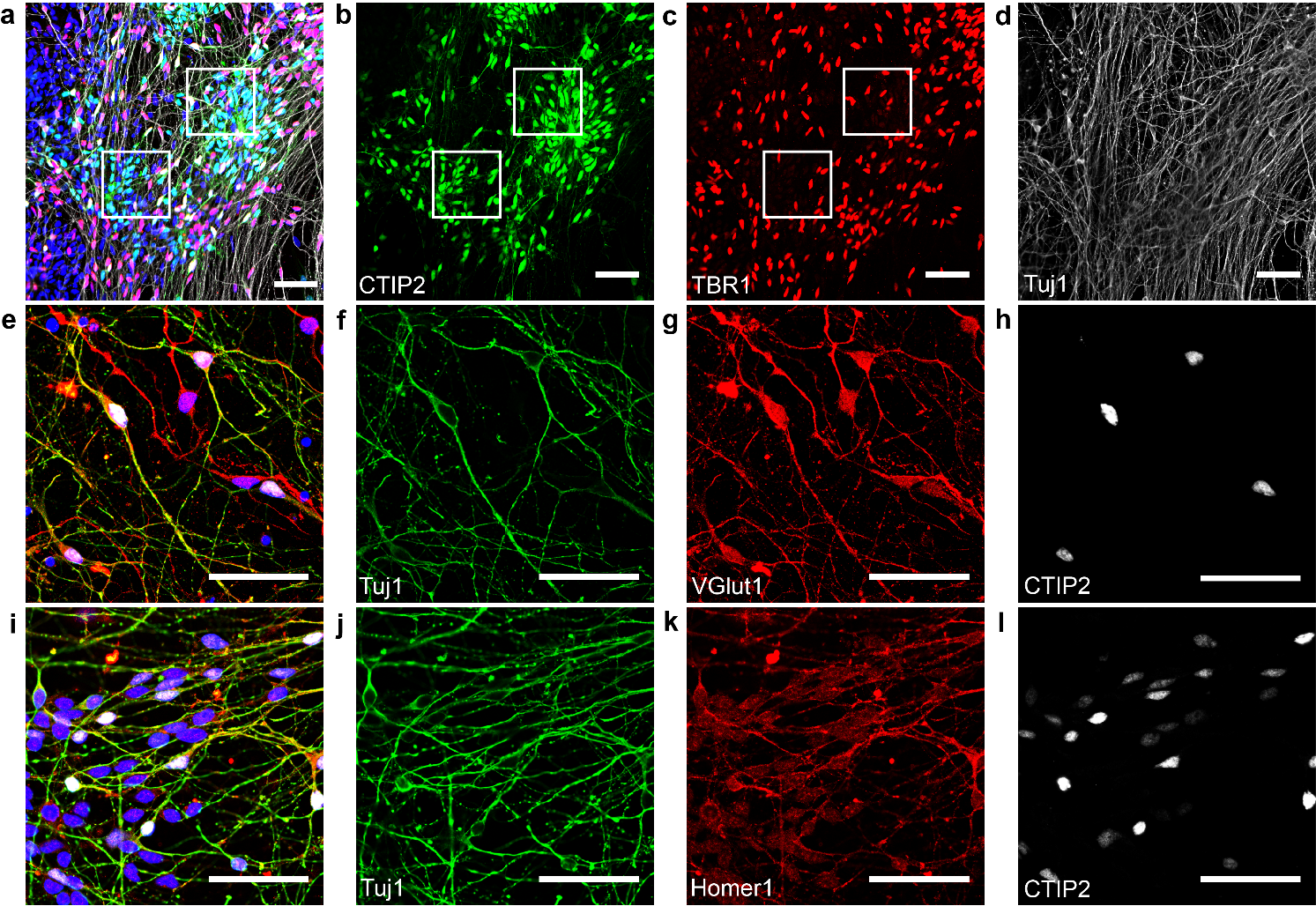


**Figure S2 CTIP2-positive PNPs at differentiation day 50-60 in vitro.** (a-d) CTIP2 and TBR1 staining at differentiation day 50,CTIP2+/TBR1- pyramidal neurons were showed in the white boxes. (e-l) Staining of the presynaptic marker VGLUT1 and the postsynaptic marker Homer1 on the CTIP2+ PNPs at differentiation day 60. bar, 50 μm.


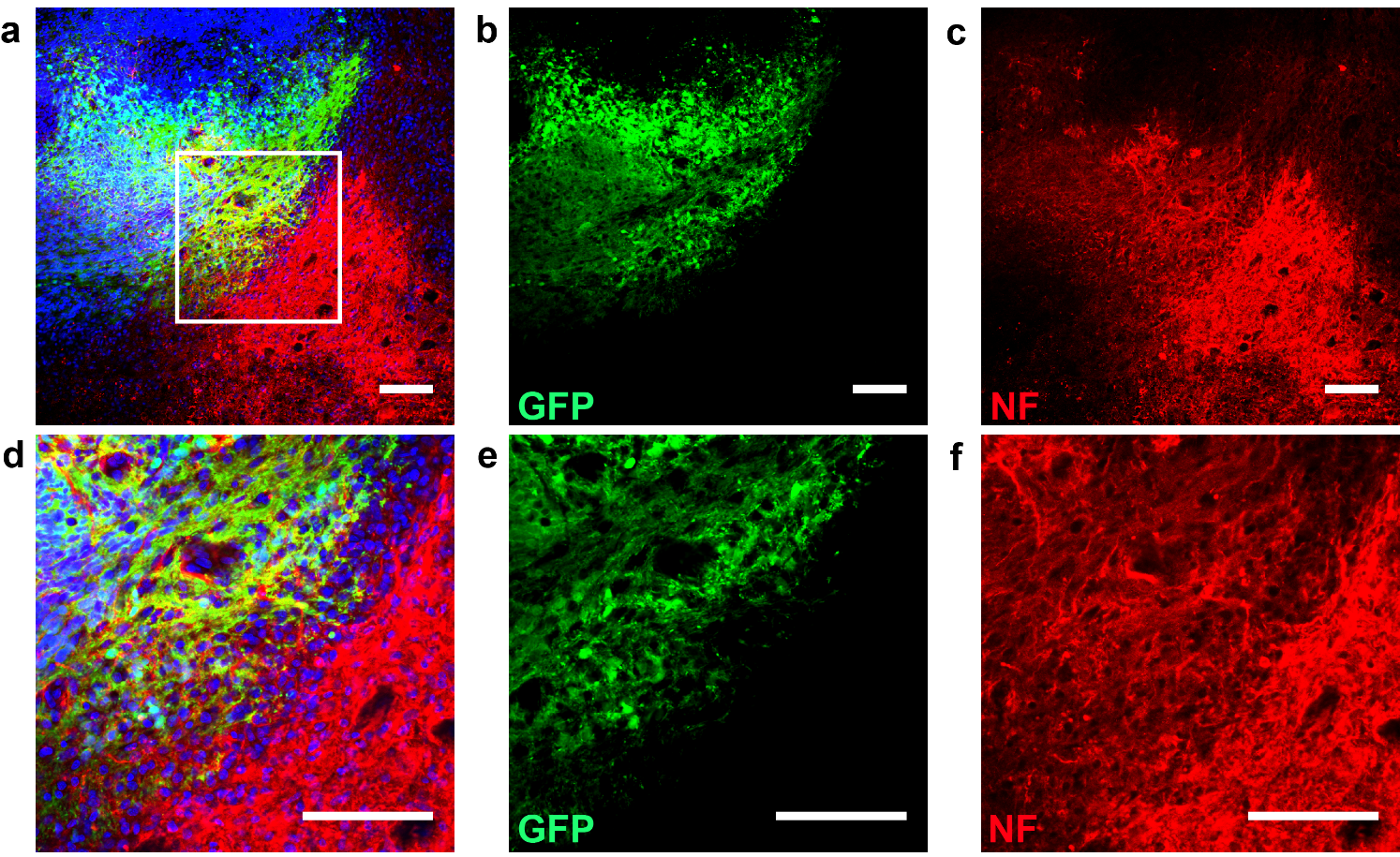


**Figure S3 Axons of rat neurons grow into the grafts 2 months following transplantation.** (a-c) The interface of grafts and rat tissue. (d-f) The view in the white box in Figure A. GFP, green; NF, red; bar, 100 μm.


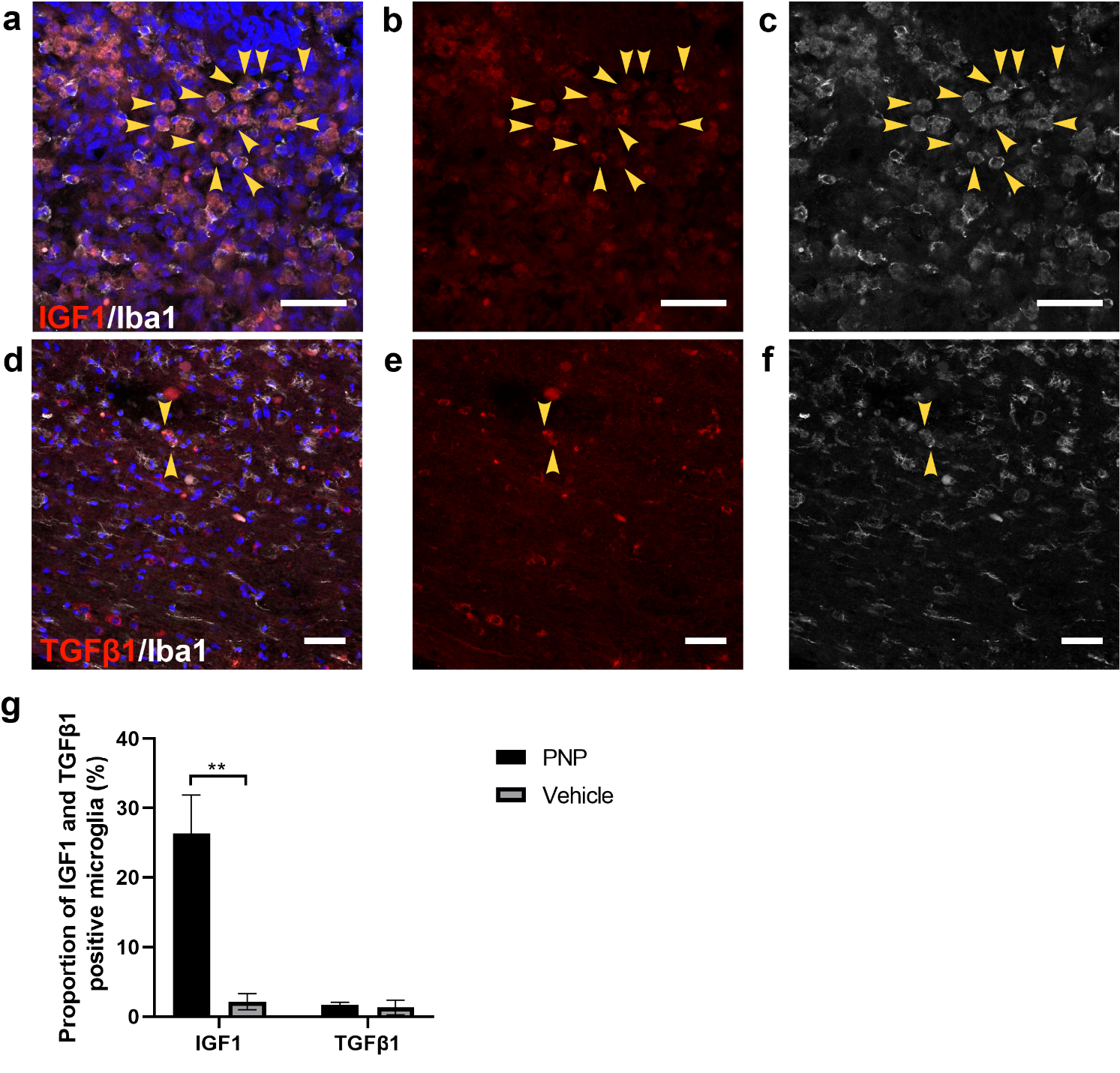


**Figure S4 IGF1- and TGF-β1- positive microglia/macrophages at the lesion site 2 w after transplantation.** (a-f) Some microglia/macrophages expressed IGF1 (a-c) and TGF-β1 (d-f) at the lesion site 2 w following transplantation. (g) The percentages of IGF1- and TGF β1-positive microglia were quantified. (n=3; * P < 0.05; ** P < 0.01; *** P < 0.001). Yellow arrows indicated the positive cells. IGF1 and TGF-β1, red; Iba1, white; bar, 50 μm.


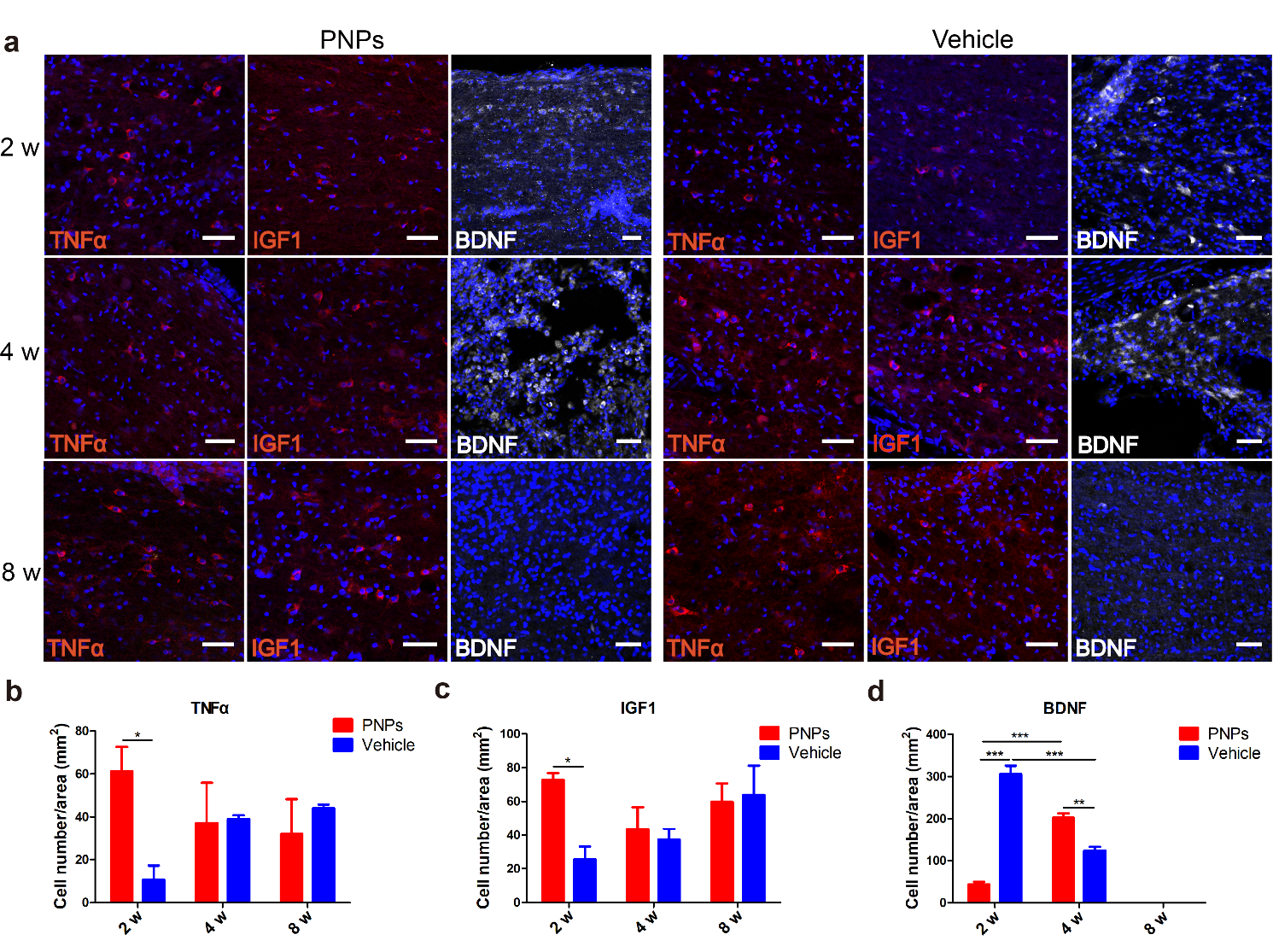


**Figure S5 Expression of the inflammatory factor TNFα, and neurotrophic factors IGF1 and BDNF at the lesion sites in SCI rats.** (a) Staining of TNFα, IGF1 and BDNF at the lesion sites in PNP and vehicle groups at 2 w, 4 w and 8 w (TNFα and IGF1, red; BDNF, white; bar, 50 μm). (b-d) The density of TNFα+ (b), IGF1+ (c) and BDNF+ (d) cells at the lesion sites (the number of positive cells divided by the area). n = 5; * P < 0.05; ** P < 0.01; *** P < 0.001.
